# Induction of Influenza-Specific Mucosal Immunity by an Attenuated Recombinant Sendai Virus

**DOI:** 10.1371/journal.pone.0018780

**Published:** 2011-04-18

**Authors:** Thuc-vy L. Le, Elena Mironova, Dominique Garcin, Richard W. Compans

**Affiliations:** 1 Department of Microbiology and Immunology and Emory Vaccine Center, Emory University School of Medicine, Atlanta, Georgia, United States of America; 2 Department of Microbiology and Molecular Medicine, University of Geneva School of Medicine, Geneva, Switzerland; Albany Medical College, United States of America

## Abstract

**Background:**

Many pathogens initiate infection at the mucosal surfaces; therefore, induction of mucosal immune responses is a first level of defense against infection and is the most powerful means of protection. Although intramuscular injection is widely used for vaccination and is effective at inducing circulating antibodies, it is less effective at inducing mucosal antibodies.

**Methodology/Principal Findings:**

Here we report a novel recombinant, attenuated Sendai virus vector (GP42-H1) in which the hemagglutinin (HA) gene of influenza A virus was introduced into the Sendai virus genome as an additional gene. Infection of CV-1 cells by GP42-H1 resulted in cell surface expression of the HA protein. Intranasal immunization of mice with 1,000 plaque forming units (pfu) of GP42-H1 induced HA-specific IgG and IgA antibodies in the blood, brochoalveolar lavage fluid, fecal pellet extracts and saliva. The HA-specific antibody titer induced by GP42-H1 closely resembles the titer induced by sublethal infection by live influenza virus; however, in contrast to infection by influenza virus, immunization with GP42-H1 did not result in disease symptoms or the loss of body weight. In mice that were immunized with GP42-H1 and then challenged with 5LD_50_ (1250 pfu) of influenza virus, no significant weight loss was observed and other visual signs of morbidity were not detected.

**Conclusions:**

These results demonstrate that the GP42-H1 Sendai virus recombinant is able to confer full protection from lethal infection by influenza virus, supporting the conclusion that it is a safe and effective mucosal vaccine vector.

## Introduction

Induction of immune responses at sites where pathogens initiate replication is crucial for the prevention of infection. The mucosal surface of the human body covers over 400 m^2^ and is the site where many pathogens such as influenza virus initiate their replication process. For influenza virus infection, antibodies are thought to mediate protection while T cells mediate the clearance of the virus [1]. Because influenza virus infection occurs first in the upper respiratory tract, induction of antibody responses in these mucosal tissues is critical for the prevention of virus infection. The role of IgA and IgG in the protection against influenza has been extensively investigated. IgA plays a dominant role in protection of the upper respiratory tract while IgG prevents lethal infection from influenza virus in the lower respiratory tract [2]. Passive transfer of polymeric IgA at levels that are naturally occurring in nasal secretions confers nearly complete protection and clearance of viruses from the upper respiratory tract, whereas much larger amounts of influenza specific IgG antibodies must be administered to provide the equivalent protection as IgA [3]. Although IgA is the predominant antibody class produced in the upper respiratory tract, passive transport of IgG antibody from the systemic circulation onto the mucosal surfaces can also enhance protection from virus infection in these tissues [4]. Together, these studies highlight the collaborative roles of IgA and IgG in the mucosal surfaces in the protection from influenza virus infections [3], [4]. The most effective approach to achieve protection from infection by influenza virus is likely to be induction of both mucosal and systemic immunity; mucosal IgA will neutralize pathogens at the site of entry and replication, and circulating IgGs that are passively transported into the lung will protect from lethal infection.

Two types of vaccines are widely used for the prevention of influenza virus infection; these are the trivalent split inactivated vaccine that is prepared by detergent solubilization of purified virus, and the cold-adapted live attenuated influenza virus (LAIV). The split inactivated vaccine is more widely used, whereas use of the live attenuated virus vaccine is limited to certain age groups. Compared to whole viral particles, the split inactivated vaccine is less immunogenic when administered at comparable doses in humans [5]. A combination of the safety attributes of the inactivated vaccine with the potent immunogenic properties of a live attenuated virus would be an advantageous property for a novel vaccine.

Recent advances in molecular genetics have permitted the development of novel virus-based vectors for the delivery of genes and expression of gene products [6], [7], [8]. These live vectors have the advantage of promoting robust immune responses due to their ability to replicate, and induce expression of genes at high efficiency. Sendai virus is a member of the *Paramyxoviridae* family, belongs in the genus respirovirus and shares 60–80% sequence homology to human parainfluenza virus type 1 (HPIV-1) [9], [10]. The viral genome consists of a negative sense, non-segmented RNA. Although Sendai virus was originally isolated from humans during an outbreak of pneumonitis [11] subsequent human exposures to Sendai virus have not resulted in observed pathology [12]. The virus is commonly isolated from mouse colonies and Sendai virus infection in mice leads to bronchopneumonia, causing severe pathology and inflammation in the respiratory tract. The sequence homology and similarities in respiratory pathology have made Sendai virus a mouse model for HPIV-1. Immunization with Sendai virus promotes an immune response in non-human primates that is protective against HPIV-1 [13], [14] and clinical trials are underway to determine the efficacy of this virus for protection against HPIV-1 in humans [15]. Sendai virus naturally infects the respiratory tract of mice and recombinant viruses have been reported to efficiently transduce luciferase, lac Z and green fluorescent protein (GFP) genes in the airways of mice or ferrets as well as primary human nasal epithelial cells [16]. These data support the hypothesis that intranasal (i.n.) immunization with a recombinant Sendai virus will mediate heterologous gene expression in mucosal tissues and induce antibodies that are specific to a recombinant protein. A major advantage of a recombinant Sendai virus based vaccine is the observation that recurrence of parainfluenza virus infections is common in humans [12], [17] suggesting that anti-vector responses are limited, making repeated administration of such a vaccine possible.

We have previously described the recovery of a mutant virus GP42-SeV containing a replacement of nucleotides 1–42 of the 3′ leader sequence encoding the virus genomic promoter (P^L^), with the corresponding sequence of the 5′ trailer sequence encoding the anti-genomic promoter (P^R^) [6]. This mutant exhibits enhanced chronic infection and reduced virus-induced programmed cell death *in vitro* [18]. In the present study, we introduced the hemagglutinin (HA) gene from influenza virus A/Puerto Rico/8/1934 (PR/8) into an intergenic sequence of GP42-SeV. This vector, designated GP42-H1, was evaluated for induction of systemic and mucosal antibodies as well as effectiveness of protection from lethal challenge with influenza virus.

## Results

### HA is expressed on the surfaces of GP42-H1 infected cells and co-sediments with virus particles

**Figure 1:**
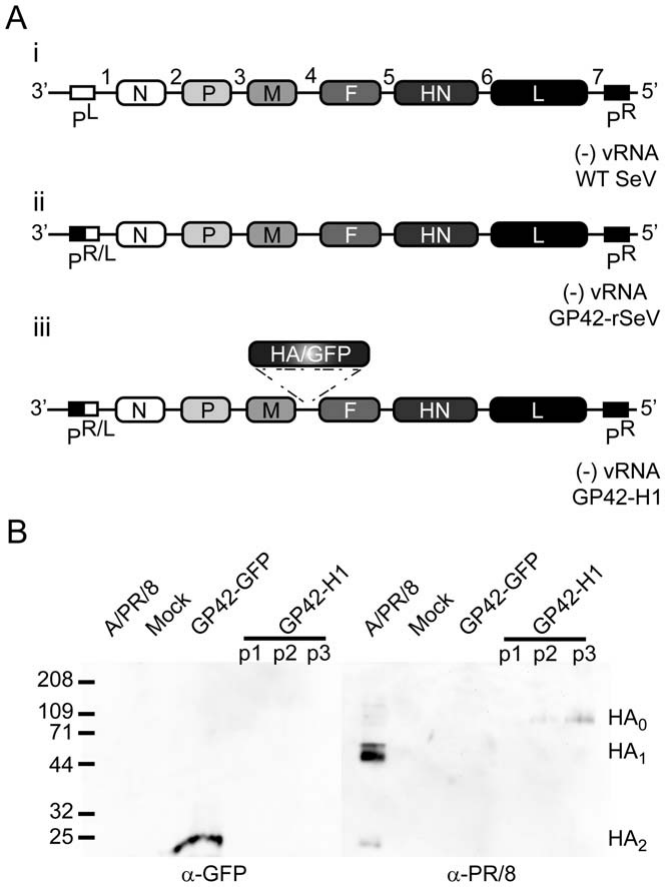
Generation of the GP42-H1 vector. A) Representation of wild-type Sendai virus gene construct (i) illustrating the major viral genes N, P, M, F, HN and L. The left promoter (P^L^) and right promoter (P^R^) function as the genomic promoter and anti-genomic promoter respectively. Seven gene boundaries that encode the conserved regulatory transcription start and transcription stop sequence are represented numerically. The mutant GP42-SeV (ii) genomic RNA is identical to WT Sendai virus with the exception of 3′ P^L^ in which 42 nucleotides of the P^L^ were replaced with the corresponding sequence from P^R^ (P^R/L^). Additional transcription start, stop, poly-adenylation sequences and a unique Mlu I restriction site were introduced into the intergenic region between the Sendai M and F genes. Using the unique Mlu I restriction site, the GFP or HA gene was inserted (respecting the rule of six) generating the recombinant Sendai GP42-GFP [19] or GP42-H1 vectors (iii). B) Recombinant GP42-H1 virus was cultured in BSR-T7 cells for three passages. Cell free supernatant containing virus suspensions were collected and used to infect CV-1 cells (refer to Materials and Methods). Proteins from GP42-GFP or GP42-H1 infected cell extracts were resolved on SDS-PAGE and screened for GFP (left) or HA (right) expression by western analysis. Mock infected cells and allantoic fluid from PR/8 infected eggs are also shown.

Using the GP42-SeV mutant that was previously described [6], the GP42-GFP vector was generated [19] and the HA gene of influenza PR/8 virus was introduced in the intergenic sequence of the viral genome between the M and the F genes to generate the GP42-H1 vector (Fig. 1A). The HA and GFP genes were stably expressed for at least three passages as determined by western analysis (Fig. 1B). Virus recovered from the third passage exhibited the highest level of HA expression and was subsequently used for further studies.

**Figure 2:**
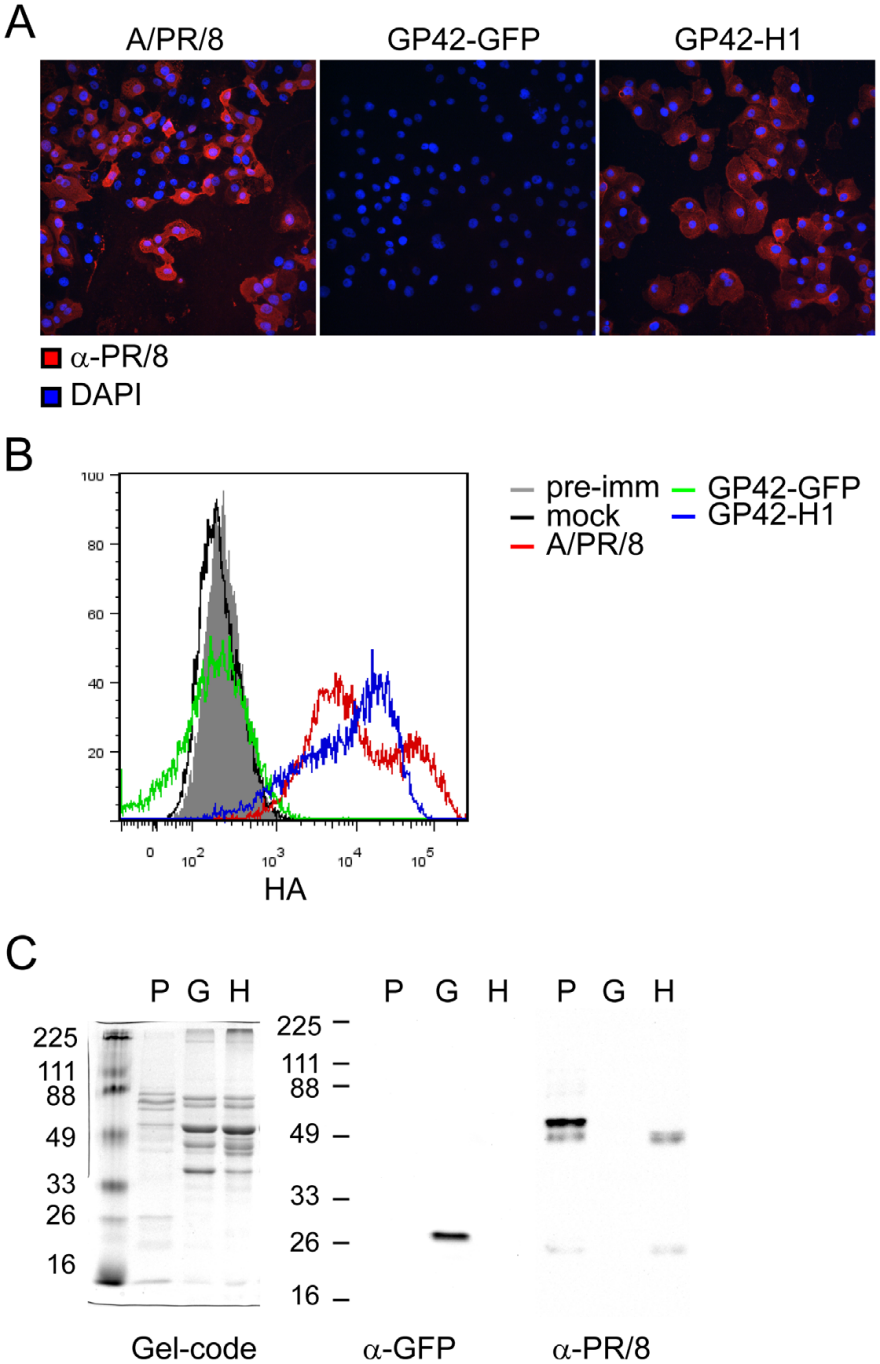
GP42-H1 vector induces expression of HA in infected cells *in vitro*. A) CV-1 cells were cultured on glass slides and infected with the indicated viruses (MOI = 10). At 20 hours post-infection, the infected cells were fixed in acetone and evaluated for HA expression by staining with mouse PR/8 anti-sera followed by TRITC conjugated anti-mouse IgG. Slides were mounted with Prolong-Antifade containing DAPI stain and visualized by immunofluorescence microscopy. B) PR/8, GP42-GFP, GP42-H1 or mock infected CV-1 cells were stained with the mouse PR/8 anti-sera. PR/8 infected cell were also stained with pre-immune sera (grey filled histogram) as a control for binding of nonspecific serum proteins. Surface bound mouse antibodies were detected with anti-mouse IgG-PE and analyzed by flow cytometry. C) Eggs were inoculated with PR/8 virus (P), GP42-GFP (G) or GP42-H1 (H) at 1 HA unit per egg. Sucrose purified recombinant viruses and allantoic fluid from PR/8 infected eggs were resolved on SDS-PAGE. SDS-PAGE gel was stained with gel-code blue (left), anti-GFP western blot (middle) and anti-PR8 western blot (right).

To further evaluate HA expression, CV-1 cells were infected with GP42-GFP, GP42-H1 or PR/8 virus at a MOI = 10. At 20 hours post infection, the cells were examined by immunofluorescence microscopy for the expression of the HA proteins (Fig. 2A). Both PR/8 and GP42-H1 infected cells exhibited uniform expression of HA protein. To identify if HA is expressed on the cell surface, GP42-H1, GP42-GFP and PR/8 infected cells were stained for surface expression of HA and analyzed by flow cytometry (Fig. 2B). As expected, the HA protein was expressed on the surfaces of PR/8 infected cells 20 hrs after virus infection. Similarly, we observed localization of the HA protein on the surfaces of GP42-H1 infected CV-1 cells.

To determine if the HA protein is associated with the GP42-H1 virus particle, virus was amplified in embryonated chicken eggs and purified through a discontinuous sucrose gradient. As shown in figure 2C, the HA protein copurified with the virions and amplification in eggs resulted in efficient cleavage of the HA_0_ precursor into the mature HA_1_ and HA_2_ polypeptides. Interestingly, GFP also co-sedimented with the GP42-GFP virus particles. These data indicate that GP42-H1 is infectious in cell lines and is competent to induce HA protein expression on cell surfaces. Co-purification of the recombinant proteins with the virus particles suggests that the HA and GFP may be incorporated into virions.

**Figure 3:**
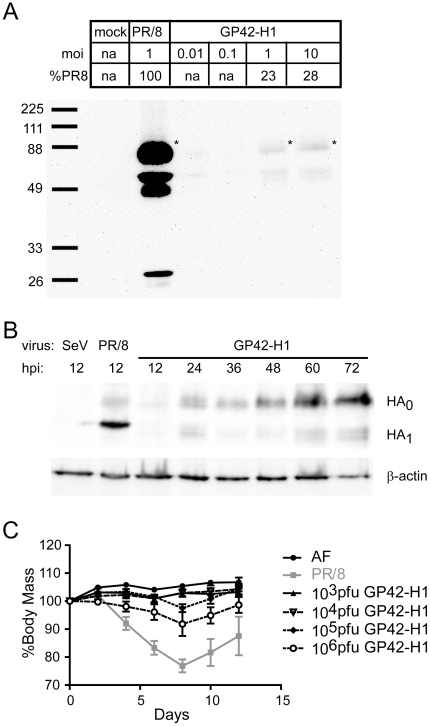
GP42 vectors induce expression of HA protein in cells and limited weight loss in mice. A) CV-1 cells were infected with PR/8 or GP42-H1 for 20 hours at the indicated MOI. SDS acrylamide gel was performed with whole cell lysates and proteins were transferred onto nitrocellulose membranes for detection of HA proteins by western analysis. All HA_0_ band intensities were calculated and expressed as a percent of PR/8 HA_0_. B) CV-1 cell were infected with WT SeV, PR/8 or GP42-H1 at MOI = 1. Whole cell lysates were collected from the infected cell at the time point indicated. Proteins were transferred onto nitrocellulose membrane and evaluated for expression of HA and β-actin. C) Mice were administered normal allantoic fluid (AF), infected (i.n.) with 1LD_50_ PR/8 or the indicated titer of GP42-H1. The percent of the initial body weight was recorded every 2 days.

To quantitate the relative expression of HA in infected cells, CV-1 cells were infected with PR/8 virus at MOI = 1 and the expression of HA was compared to cells infected with GP42-H1 at MOI of 0.01 to 10. Whole cell lysates were collected from infected cells 20 hours post infection and HA protein expression was evaluated by western immuno-blot. The expression of HA increased directly with increasing virus MOI (Fig. 3A); however, when infected at equivalent MOI, the expression of HA induced by GP42-H1 was approximately 23% compared to PR/8 infection (Fig. 3A and Table 1). Compared to wild-type (WT) Sendai virus, GP42-H1 grows more slowly [18] which may contribute to reduced expression of HA. To evaluate the kinetics of the expression of HA, CV-1 cells were infected with GP42-H1 and whole cell lysates were collected every 12 hours, for 72 hours. Protein expression was evaluated by western blot (Fig. 3B) and integrated density values for actin, HA_0_ and HA_1_ band were recorded (Table 2). At 12 hours post inoculation, HA was not detected in the lysate of GP42-H1 infected cells as opposed to cells that were inoculated with PR/8 where the expression of the HA_0_ and HA_1_ was 21% compared to actin protein. However, at 72 hpi, the expression of HA from cells inoculated with GP42-H1 was 22% compared to actin, a level comparable to that observed in PR/8 inoculated cells.

**Table 1:** Comparison of the expression of HA in PR/8 infected cells and GP42-H1 infected cells.

	Mock	PR/8	GP42-H1			
**MOI**	N/A	1	0.01	0.1	1.0	10
**PFU**	N/A	5.4×10^6^	5.4×10^4^	5.4x10^5^	5.4×10^6^	5.4×10^7^
**IDV**	N/A	284580	N/A	N/A	65844	79236
**% PR/8**	N/A	100	NA	N/A	23.14	27.84

CV-1 cells were infected with PR/8 virus or GP42-H1 vectors at the indicated MOI. The numbers of plaque forming units from the corresponding viruses were estimated based infectivity assay. 20 hours post infection, the cells were lysed and SDS-PAGE was performed on the whole cells lysates. The proteins were transferred to nitrocellulose membrane and PR/8 immuno-blot was performed. Flourchem FC2 software was used to measure the pixel densities from the HA_0_ bands and pixel densities were translated to integrated density values (IDV) by the software.

**Table 2:** Evaluation of virus-induced HA expression in CV-1 cells.

Virus	WT SeV	PR/8	GP42-H1					
**HPI**	12	12	12	24	36	48	60	72
**HA_0_**	60160	85728	62416	116560	87984	125584	128592	178224
**HA_1_**	70688	301552	54144	72192	56400	62416	72944	82720
**actin**	1888050	1825422	1857657	2303421	1903707	2223294	2110932	1199142

The western blot from figure 3b was evaluated by FluorChem western blot imaging system and the integrated density values were measure after 5, 15 and 30 sec exposure. Depicted are the density values following a 30 second exposure.

### The GP42-SeV vector exhibits an attenuated phenotype *in vivo*

Infection of mice with WT Sendai virus induces severe respiratory tract pathology and infection with 1,000 WT pfu can lead to weight loss while infection with 1 million pfu is lethal to mice [20]. *In vitro*, visible signs of cell stress such as chromatin condensation are observed in cells that are infected with WT Sendai virus and most cells die within 72 hours after infection. In contrast to WT Sendai virus, cells that are infected with GP42-SeV exhibit little cytopathic effect and significant reduction of apoptosis is observed [18]. This has been attributed to a reduction of virus-induced programmed cell death, a feature that is mediated by the 42 nucleotide 3′ leader sequence that is deleted in the mutant GP42-SeV virus and replaced by the corresponding 5′ trailer sequence. We predicted that the replacement of the 3′ leader sequence may also contribute to an attenuation of the virus *in vivo*. To test this, mice were immunized (i.n.) with the GP42-H1 vector in doses ranging from 10^3^–10^6^ pfu and evaluated for changes in body weight and other signs of illness such as ruffled fir and hunched posture. GP42-H1 infected mice did not show visible signs of disease whereas mice that were infected with 250 pfu (1LD_50_) of influenza PR/8 exhibited ruffled fir and hunched posture within three days post-infection. By 4–6 days post infection, several mice that received PR/8 had lost at least 25% of the initial body weight and were euthanized. In contrast, mice that received 10^5^ pfu GP-42-H1 (i.n.) exhibited only 5–10% loss of the initial body weight (Fig. 3C). Less than 5% body weight loss was observed in mice that were infected with 10^4^ pfu of GP42-H1 while mice infected with 10^3^ pfu GP42-H1 did not exhibit any weight loss. All mice infected with these dose ranges of the GP42-H1 vector survived. Only after infection with 10^7^–10^8^ pfu did mice show visible signs of illness or lethality (data not shown). These data strongly support the conclusion that the GP42 vector is attenuated and is potentially safer as a live vector than wild type Sendai virus.

### Immunization with GP42-H1 induces HA specific antibodies in the blood and mucosal surfaces

**Figure 4:**
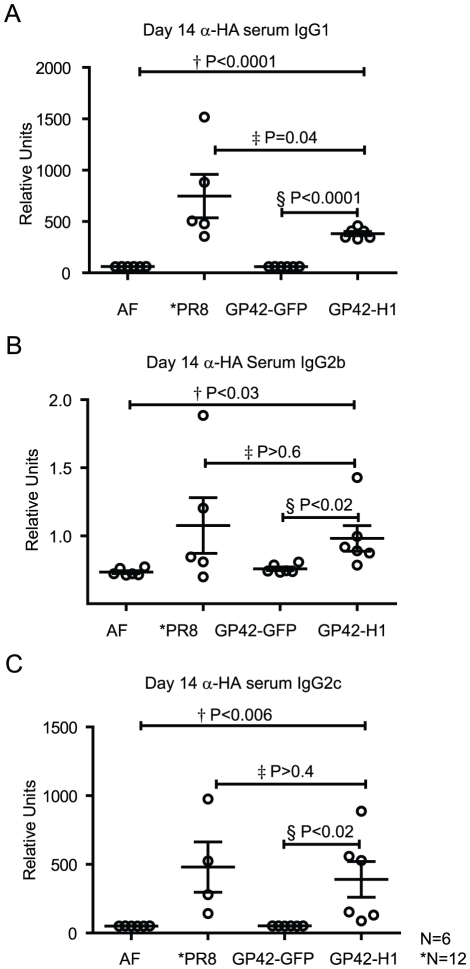
GP42-H1 induces HA-specific antibodies in sera. Mice were administered AF, 1LD_50_ PR/8 or 10^3^ pfu of either GP42-GFP (vector control) or GP42-H1. Blood was collected 14 days after infection and sera were assayed by ELISA for A) PR/8-specific IgG1 B) PR/8-specific IgG2b and C) PR/8-specific IgG2c antibodies. Student's T tests were performed between each group (n = 6 for all groups except the PR/8 group where n = 12). The P values between each group are indicated. Experiments were repeated 2 times with similar results.

HA-specific antibodies are critical for protection from infection by influenza virus. Assays such as HAI and microneutralization are commonly used to detect and quantitate the antibody titer and high HA-specific titers are a standard correlate for protective immunity in humans. To evaluate the immune response, mice were administered pathogen free AF (i.n.) as a negative control, or were immunized (i.n.) with 1,000 pfu of GP42-GFP as a vector control or 1,000 pfu of GP42-H1. The GP42-GFP and GP42-H1 vectors are replication competent and able to infect brochoepithelial cells; therefore, we tested for HA-specific antibodies in sera from mice that were infected with these vectors and compared the titers to antibody titers from animals that were infected with 250 pfu (1LD_50_) of live PR/8 virus. Blood was collected and circulating PR/8-specific IgG1, IgG2b and IgG2c were measured by ELISA (Fig. 4A–C). C57Bl/6 mice express the IgH-1^b^ allelic gene and therefore express the IgG2c isotype rather than IgG2a [21], [22]. Immunization with the GP42-H1 vector induced PR/8-specific antibody titers closely resembling those induced by infection with PR/8 virus. The antibody response was dominated by very similar concentrations of IgG1 and IgG2c while low titers of PR/8 specific IgG2b were detected in the serum.

**Figure 5:**
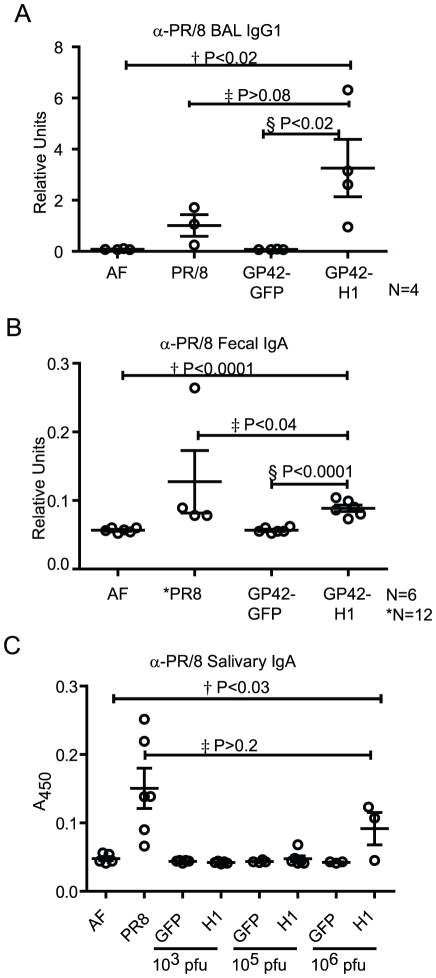
GP42-H1 induces HA-specific antibodies at mucosal sites. A) Mice were infected with 0.25 LD_50_ (63 pfu) of PR/8 or 10^3^ pfu of either GP42-GFP or GP42-H1. At 20 days post infection, mice (n = 4) were sacrificed and BAL fluid was collected and assayed for PR/8-specific IgG1 antibodies. B) At 14 days post-infection, fecal pellet suspensions and C) saliva were collected from the mice (n = 6 for all groups except PR/8 where n = 12) described in figure 4 and assayed for the presence of PR/8 specific IgA antibodies. Shown are representative data from one of three independent experiments.

To evaluate if HA-specific antibodies were also present at the mucosal surfaces, PR/8-specific antibodies were measured in brochoalveolar lavage fluid (BALF), fecal pellet extract and saliva (Fig. 5A–C). Two weeks post infection, PR/8-specific IgA was detected in fecal pellet extracts of mice infected with 1LD_50_ of PR/8 and 1,000 pfu of GP42-H1 but not in extracts from mice infected with 1,000 pfu of GP42-GFP or mice that received AF. PR/8-specific IgA was present in the saliva of mice that were infected with PR/8 and mice that were infected with 10^6^ pfu of GP42-H1 but not in the saliva from mice that received 10^3^ pfu GP42-H1, GP42-GFP or AF. BALF were evaluated for HA-specific antibodies at 7–20 days post infection. Very low concentrations of HA-specific mucosal antibodies were detected in the first two weeks post infection (data not shown). To assure survival of the PR/8 infected mice beyond 1 week post infection, mice were infected with 65 pfu (0.25 LD_50_) of PR/8 virus, a dose that caused minor weight loss in mice but did not cause mortality up to 20 days post infection. By three weeks post infection, HA-specific IgG antibodies were detected in the BALF. Although IgA antibody titers were below the threshold of detection in the BALF of PR/8 and GP42-H1 infected mice, PR/8-specific IgG1 was detected in the BALF of both PR/8 and GP42-H1 infected mice but not in GP42-GFP vector control or negative control BALF. These results demonstrate that infection with the GP42-H1 vector through the i.n. route induces HA-specific antibodies. These antibodies were present in both the circulating blood as well as in the mucosal secretions including saliva, fecal pellet extracts and BALF. Furthermore, infection by GP42-H1 did not cause weight loss unlike infection with influenza virus.

### Immunization with GP42-H1 induces antibodies that inhibit hemagglutination and neutralize virus replication *in vitro*

**Figure 6:**
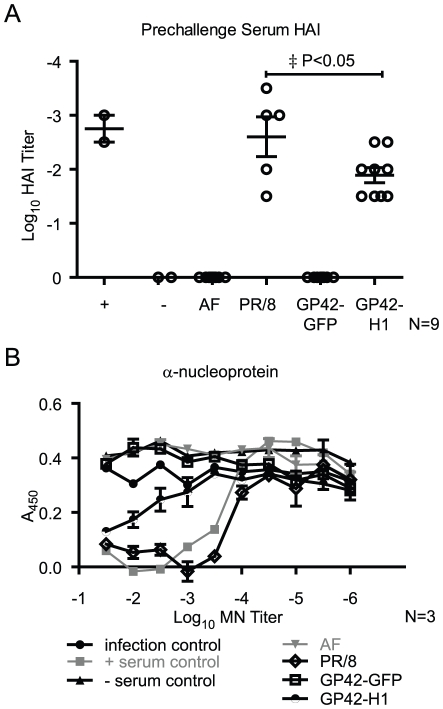
GP42-H1 induces antibody responses that block receptor binding and neutralize virus infection *in vitro*. Blood was collected from mice six weeks after the mice were administered (i.n.) AF, 250 pfu PR/8, 10^3^ pfu GP42-GFP or 10^3^ pfu GP42-H1. A) Serum was assayed for inhibition of PR/8 dependent hemagglutination. Each data point represents the highest dilution of serum exhibiting complete inhibition of PR/8 mediated hemagglutination collected from individual mice. The lines depicts the means ± standard errors of the mean. B) Serially diluted sera were tested for inhibition of PR/8 infection of MDCK cells by micro-neutralization test. Each data point represents the mean ± the standard errors of the mean of three randomly chosen samples from each experimental group. Values depicted are the optical density subtracted from background reading containing only cells. LogEC_50_ of PR/8 is -3.93. LogEC_50_ of GP42-H1 is -2.19. P<0.0001.

The above results demonstrate that infection with GP42-H1 induces antibodies specific to the HA protein of PR/8 influenza virus. To evaluate if the antibodies induced by the infection with GP42-H1 can prevent virus attachment to cell surface receptors, blood was collected from mice at 6 weeks post-infection (one week prior to challenge) and the serum was evaluated for inhibition of erythrocyte hemagglutination (HAI) and inhibition of influenza virus infection of MDCK cells. Sera from mice infected with GP42-H1 vectors exhibited mean HAI titers of Log -1.67, or 46.5 reciprocal dilution (Fig. 6A). HAI titers greater than 40 are correlates of protection from influenza infection in humans. Serum antibodies were further evaluated for neutralization activity (Fig. 6B). GP42-H1 vector-induced antibodies were functionally competent to inhibit influenza virus infection of cultured MDCK cells albeit with lower titers than antibodies from mice that survived infection by influenza virus.

### GP42-H1 immunization protects mice from lethal challenge with PR/8 virus

**Figure 7:**
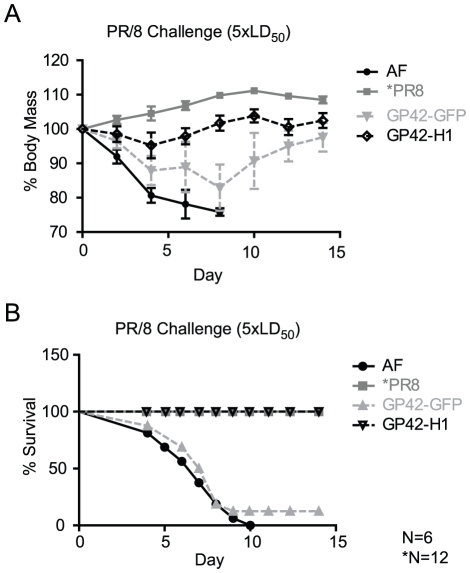
GP42-H1 protects mice from lethal challenge with PR/8 virus. Mice that were administered AF, infected with 1LD_50_ of PR/8 virus or immunized with 1,000 pfu of GP42-GFP, or 1,000 pfu GP42-H1 (i.n.) were challenged (seven weeks post-immunization) with 5LD_50_ of PR/8 virus (i.n.) A) Body weight was recorded over 14 days and the percent of the body weight at the time of challenge is shown. B) Animals were monitored for 14 days post challenge and morbidity is recorded. Depicted are the percentages of mice that survived the virus challenge.

Antibodies induced by immunization with the GP42-H1 vector effectively neutralized virus infection *in vitro*. To test the efficacy of GP42-H1 to induce protection *in vivo*, mice were challenged with a lethal dose of mouse adapted PR/8 virus (i.n.). Mice were administered (i.n.) AF from pathogen free eggs (negative control), 1,000 pfu of GP42-GFP (vector control), or 1,000 pfu of GP42-H1. The mice were challenged (i.n.) seven weeks later with 1250 pfu (5LD_50_) of PR/8 virus and monitored for visible signs of illness and changes in body weight for two weeks. Three to four days post challenge, the negative control mice that were administered AF exhibited signs of illness that included hunched posture and ruffled fir. Mice that were immunized with the GP42-GFP vector control also exhibited similar signs of morbidity between 4-6 days post-challenge. In contrast, mice that were immunized with GP42-H1 did not show signs of illness. Evaluation of body weight also indicated that GP42-H1 immunized mice were protected from PR/8 challenge (Fig. 7A). Of the six mice that were immunized with GP42-H1, only two lost weight within two to four days after i.n. challenge. Over four days, these mice lost an average of 15% of their starting weight from the time of challenge. By ten days post challenge, these mice completely recovered and regained the weight that they lost. The remaining four mice that were immunized with GP42-H1 exhibited complete protection from the challenge and no weight loss was observed. These data confirm that immunization with the GP42-H1 vector resulted in protective immunity to homologous influenza virus challenge. In contrast, administration of AF or immunization with GP42-GFP did not protect the mice from challenge; these mice rapidly lost 25% of the starting body weight and were subsequently euthanized. The response induced by a single immunization with GP42-H1 was sufficient to protect 100% of mice from death by PR/8 infection (Fig. 7B) in contrast to a 0% survival rate from mice administered AF and 12.5% survival rate from mice inoculated with GP42-GFP.

**Figure 8:**
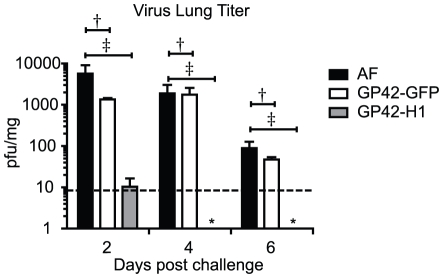
GP42-H1 infection promotes rapid clearance of PR/8 virus challenge. Animals were challenged with 5LD_50_ of PR/8 virus and the lungs were aseptically removed. The lungs were homogenized and cell free suspensions were collected and assessed for lung virus titers by plaque assay. Data are depicted as the mean pfu per mg of lung homogenate + standard errors of the mean. N = 4. Samples marked by an asterisks (*) did not form plaques and were below the threshold of detection (dashed line). ^†^ P>0.05 ^‡^P<0.01.

Evaluation of lung viral titers revealed that at two days post-challenge, lungs from mice that received AF contained a virus titer of greater than 5,700 pfu/mg of lung weight. The virus titer from lungs of GP42-GFP immunized mice was 1,350 pfu/mg. In contrast to control mice, the lungs of mice that received GP42-H1 contained a mean virus titer of approximately 10 pfu/mg. This is nearly 1,000-fold lower than the virus titer that was observed in lungs of mice that received AF (P<0.01) and 100-fold lower than the lung virus titer of the GP42-GFP vector control (Fig. 8). By four days post-challenge, GP42-H1 immunized mice completely cleared PR/8 virus from the lung. These data demonstrate that immunization with GP42-H1 reduced influenza virus replication during challenge and/or promoted rapid clearance of the virus upon lethal challenge.

## Discussion

Using a GP42-SeV mutant that exhibits reduced cytopathic effects and attenuated phenotype in mice, we generated a recombinant Sendai virus vector that mediates the heterologous expression of the HA gene of PR/8 influenza virus in infected cells. Following intranasal immunization with 1,000 pfu of GP42-H1, PR/8 specific IgA and IgG antibodies were induced and detected in the blood and fecal pellet extracts. PR/8 specific IgA was also detected in saliva upon immunization with 10^6^ pfu of GP42-H1. We demonstrated that antibodies in the serum of GP42-H1 immunized mice inhibited virus mediated red blood cell agglutination and also inhibited virus infection of MDCK cells. Mice that were immunized with GP42-H1 were protected from homologous lethal challenge with PR/8 virus whereas mice that received GP42-GFP vector control or allantoic fluid alone, succumbed to lethal infection.

The concentration of the HA-specific antibodies in the serum and fecal pellet extract of GP42-H1 immunized mice were moderately lower than the antibody titers from PR/8 infected mice; however, these differences were not statistically significant. In contrast, the HAI titer and micro-neutralization titer from GP42-H1 immunized mice were significantly lower than titers from PR/8 infected serum. The lower titer associated with GP42-H1 is likely due to the induction of only HA-specific antibodies whereas immunization with PR/8 induced other influenza virus-specific antibodies such as anti-NA antibodies. We predict that the concentration of anti-HA antibodies can be increased by optimizing the GP42-H1 virus immunizing dose; however, immunization with 1,000 pfu for GP42-H1 was sufficient to induce protection. We also tested lower immunizing doses and found that immunization with 100 pfu of GP42-H1 was also protective against homologous lethal challenge (data not shown). These data demonstrate that low doses of the GP42-H1 vector are competent to induce protective immunity against homologous influenza challenge in the *murine* model. Because the vector is replication-competent, we hypothesize that it will also promote induction of cell mediated immunity. This property may contribute to the observed low dose required for protection.

When GP42 vectors were administered (i.n.) to mice, minimal weight loss was observed. Furthermore, GP42-H1 was competent to induce HA-specific antibodies which were detected in the mucosal secretions including IgA antibodies in saliva and fecal pellet extracts and IgG antibodies in BALF. Previous findings have demonstrated the protective role of IgG antibodies in the lower respiratory tract [2], [3]. Although PR/8 specific IgA were below the threshold of detection in BALF, these antibodies were detected in fecal pellet extracts and saliva. These observations demonstrate that the recombinant virus is competent to induce antibody responses in the mucosal tissues. The low concentration of IgA observed in the saliva and BALF is not unexpected as measurement IgA is highly variable in saliva; furthermore, IgA antibodies are found at low concentration in the BALF as opposed to the more abundant IgG antibodies [2], [23]. Previous findings have demonstrated the critical role of IgA in the protection of the upper respiratory tract and IgG protection in the lower respiratory tract [3]. The induction of antibodies in the mucosal tissues by the recombinant GP42-H1 vector is promising for the prevention of infection by pathogens such as influenza virus. In addition to promoting protection in the upper respiratory tract, IgA antibodies exhibit broad specificity across heterosubtypic viruses [24] and play a critical role in heterosubtypic protection [23], [25].

The incorporation of the Fusion (F) and Hemagglutinin-Neuraminidase (HN) proteins of Sendai virus is mediated by the cytoplasmic domains of the respective proteins [26]. Sendai as well as influenza viral glycoproteins are targeted to the apical surfaces of infected cells where virus assembly occurs [27], [28]. The HA protein associates with sphingolipids and cholesterol rich lipid rafts via the transmembrane domain and the association mediates viral budding [29], [30], [31]. Since the HA alone can mediate budding [32], the protein may also be actively incorporated into virus particles or virus-like particles through mechanisms that are independent of Sendai virus mediated pathways. This may play a role in the recovery of HA proteins with virus particles from sucrose density gradients.

At 20 hours post-infection, GP42-H1 induced expression of HA in infected cells was observed to be approximately 20% of the level observed in PR/8 infected cells. The HA protein concentration correlated with the multiplicity of virus used to infect cells. The GP42-SeV vector grows at a slower rate as compared to WT Sendai virus or influenza virus (Fig. 3B,) which may contribute to reduced expression of HA at the early stage of virus replication; however, we observed a significant increase in HA expression over 72 hours after infection.

A number of other recombinant vectors have been developed as novel vaccine strategies. One example is recombinant adenovirus, which mediates high expression of heterologous genes; however it is limited by anti-vector immune responses which can prevent repeated administration [33]. Several recent studies have explored recombinant Newcastle Disease virus (NDV) to mediate the expression of influenza virus-specific genes in poultry [8], [34], [35]. The recombinant NDV is similar to Sendai virus based vectors and considered a potential bivalent vaccine because it induces immunity specific to influenza as well as NDV. Intranasal delivery of NDV vectors to ferrets and non-human primates is capable of inducing HA-specific immunity at the mucosal surfaces [36], [37]. Other vectors include parainfluenza virus 5 (PIV5) which bear more resemblance to the GP42 recombinant SeV vector. Similar to SeV, PIV5 is not known to cause clinical symptoms in humans; however, this virus may promote canine kennel cough in companion animals [38]. PIV5 mediated expression of HA can induce protective responses against homologous influenza virus challenge; however, a 10-fold greater dose of the recombinant PIV5 was used for immunization to achieve similar protection as GP42-H1 [39].

Sendai virus can readily infect respiratory tract epithelial cells in non-human primates [16]. Intranasal administration of Sendai virus to humans is well tolerated and is capable of inducing antibodies that exhibit cross-reactivity to the closely related HPIV-1 [15]. In studies that examined the efficiency of Sendai vector transduction following repeat administration compared to a single administration, a reduction in the expression of the heterologous gene was observed [16]. However, the authors analyzed the efficiency of gene expression following a second administration 21 days after the initial administration of the recombinant virus. Longer term studies are necessary to evaluate the transduction efficiency following multiple administrations of Sendai vectors. Moreover, mice mount a robust systemic immune response to Sendai virus and can rapidly clear the virus following a second infection, making the *murine* model less suitable for studies of anti-vector responses. In contrast, human parainfluenza viruses are well known to cause recurrent infections, and protective immunity is of short duration [12], [17]. Antigenic mapping of HN, F and NP proteins of HPIV-1 [40] as well as sequence analysis of HN genes [41], [42] from clinical isolates taken over a 25 year period reveal that mutations were non-cumulative, changes in the sequence and antigenic epitopes were slow and several co-circulating strains were observed during a single season. A recent study also highlights that children with severe respiratory infections have serial infections with the same strain of parainfluenza virus as opposed to persistent infections [43]. These data are highly indicative of virus infections that are under low immunological pressure. Based on these observations it is likely that recombinant Sendai virus could be successfully used in humans for annual seasonal influenza vaccination with limited interference by anti-vector immunity.

A major advantage of the GP42 vector is the copy-back mutation resulting in the duplication of the 5′ anti-genomic promoter and a partial deletion of the genomic promoter resulted in a non-defective virus with two strong anti-genomic promoter; as a result, the virus in noncytopathic in infected cells and attenuated in mice. While the GP42 promoter architecture renders the virus less pathogenic, this virus is dominant over WT viruses and out competes WT viruses in co-infections [6]; as a result, the risk for reversion, recombination, contamination or interference with another paramyxovirus is highly unlikely. Early during infection, the virus growth kinetics is attenuated and produces lower levels of viral proteins including those dedicated to counteract innate immunity. Moreover, increased production of the 5′trailer RNA may also serve as a pathogen associated molecular pattern generating a potent live attenuated vector with natural adjuvant properties. Similar to the cold-adapted live attenuated influenza virus vaccine, GP42-H1 can induce local mucosal immune responses at the site of viral entry; however, the recombinant GP42 vector has advantages over the current LAIV vaccine. LAIV introduces replication competent influenza virus that potentially can exchange genome segments in the event of a co-infection, resulting in reassortants. In contrast, as a nonsegmented virus, this safety concern is eliminated with GP42-H1. Moreover, the GP42 Sendai virus vector has potential as a generalized mucosal vaccine vector. Although influenza was used in this study, introduction of the envelope protein of other viruses such as HIV may potentially generate other mucosal vaccines. He we have shown that GP42 vector is competent to induce local immunity at the mucosal surfaces as well as systemic immunity. Pathogens such as HIV which initiate infection at the mucosal surfaces and escape to initiate a secondary systemic infection, illustrate a critical feature that needs to be addressed in the next generation of vaccines.

The rapid production and dissemination of vaccines for potential pandemics such as H5N1 is of the greatest importance. As demonstrated in the mouse model, low doses of the vector can protect mice from lethal challenge with influenza virus. With the development of high capacity mammalian cell bioreactors, the relative ease of propagation of the virus in cell culture without the need for rigorous purification and inactivation of the virus makes this an attractive alternative to production of inactivated vaccines in eggs. Also, propagation of the vector in eggs yields virus titer approaching 10^10^ infectious particles per individual egg [44]. This high yield, ease of producing vaccines in fertilized chicken eggs and the low cost of production, provide an attractive approach for rapid and economical production of influenza vaccines in the event of a new global pandemic.

## Materials and Methods

### Ethics Statement

This study was carried out in strict accordance with the recommendations in the Guide for the Care and Use of Laboratory Animals of the National Institutes of Health. Mice were sterile housed and treated according to Emory University (Atlanta, GA) guidelines and all animal studies were approved by the Emory University Institutional Animal Care and Use Committee (Emory animal welfare assurance number A3180-01).

### Virus

Influenza A/PR/8 (H1N1) virus was obtained from the American Type Culture Collection (Manassas, VA) and propagated in 10 day old specific pathogen-free embryonated chicken eggs. Three days post inoculation, influenza virus was collected from allantoic fluid that was precleared by centrifugation. Influenza virus was purified as previously described [45]. In brief, PR/8 virus from allantoic fluid was concentrated by cross-flow filtration through a 300,000 NMWC hollow fiber using the QuickStand system (GE Healthcare; Piscataway, NJ). Influenza virus was purified by ultracentrifugation through a discontinuous 60%–30%–15% sucrose gradient and the sucrose was removed by dialysis in PBS. Virus titer was determined by plaque assay as previously described [46] and purity was confirmed by gel-code blue (Thermo Scientific, Rockford, IL) stain of SDS-gels. Mouse adapted A/PR/8 virus was propagated in the mouse lung for at least 8 passages. Mouse adapted PR/8 was collected from lung suspensions and all virus preparations were stored at −80°C.

Sendai virus was amplified in ten day old specific pathogen-free embryonated chicken eggs and harvested from the allantoic fluid three days post-inoculation. Sendai virus titers were determined by measuring plaque formation on infected CV-1 (ATCC CCL-70) cell monolayer. Sendai virus was serially diluted and 0.5 ml of the virus dilutions were adsorbed onto CV-1 cell monolayer for 30 minutes at 33°C. Unbound Sendai virus was removed by washing in PBS and the cells were overlaid with 1% agar in DMEM containing 100 units/ml penicillin, 100 µg/ml streptomycin, 25 mM HEPES buffer, 2 mM L-glutamine, 160 µg/ml diethylaminoethyl dextran, 1X non-essential amino acids and 1 µg/ml of L-1-Tosylamide-2-phenylethyl chloromethyl ketone (TPCK) treated trypsin (Sigma Aldrich; St. Louis, MS). Plaques formed 3–4 days post inoculation and the cells were fixed in 0.25% glutaraldehyde solution. The agar was removed and the plaques were visualized by staining the cell monolayer with 0.1% neutral red.

### Animals

C57BL/6 mice were purchased from Jackson Laboratory (Bar Harbor, Maine) at 6–8 weeks of age. Blood was collected from the lateral saphenous vein and allowed to clot overnight at 4°C. Serum was collected from the clotted blood by centrifugation. Salivation was induced by administering 2 µg of carbamylcholine intraperitoneally (i.p.). Fecal pellet extracts were collected by weighing fresh fecal pellets and PBS was added to a final concentration of 200 mg/ml. The fecal pellets were resuspended by vigorous vortexing followed by centrifugation and the suspension was collected. BALF was collected from mice anesthetized under Ketamine (93 mg/kg body weight) and Dormitor (1.25 mg/kg body weight). The chest cavity was opened and blood was perfused from the lung by injecting PBS into the right ventricle. A catheter was inserted into the trachea and ligated. The lung was flushed with 0.9 ml PBS to collect the BALF. All samples were stored at −20°C until further use.

For immunizations, mice were lightly anesthetized under isoflurane gas and administered (i.n.) a 20 µl volume of allantoic fluid (AF) collected from eggs that were inoculated with PBS, one 50% Lethal Dose (LD_50_) of PR/8 virus, 0.25 LD_50_ of PR/8 virus, 1,000 pfu of GP42-GFP, or 1,000 pfu of GP42-H1. The LD_50_ of PR/8 virus was determined using the method described by Reed and Muench [47] where one LD_50_ was found to equal 250 pfu of PR/8 virus. Seven weeks post immunization, mice were challenged (i.n.) with 5 LD_50_ of mouse adapted PR/8 virus (1250 pfu). All animals were housed in barrier facilities and maintained under protocols approved by the Institutional Animal Care and Usage Committee (IACUC) at Emory University.

### Generation of recombinant GP42-H1 virus

Generation of the GP42-GFP virus was previously described [19]. GP42-H1 virus was prepared similarly to GP42-GFP. In brief, a transcription start, transcription stop, poly-adenylation sequences and a unique Mlu I restriction site were introduced into the intergenic region between the Sendai M and F genes of the pSP65 plasmid encoding the GP42-SeV viral cDNA. The hemagglutinin (HA) gene of PR/8 was amplified from the pCI vector encoding the HA gene (a gift from the Center for Disease Control) using the forward primer 5′-atatacgcgtgccatgaaggcaaacctactgg-3′ and reverse primer 5′-atatacgcgtctgtcagatgcatattctgcactgc-3′. Using the unique Mlu I restriction site, the HA gene of PR/8 was introduced into to the intergenic sequence in between the M and the F genes as an additional gene. BSR-T7 cells that constitutively express T7 polymerase were transfected with 10 µg of pSP65 plasmid encoding the full-length anti-genomic viral RNA containing the HA gene, pTM1 plasmids [48] encoding the Sendai virus polymerase genes L (0.5 µg), P that contains a C stop mutation resulting in the loss of expression of the C protein (1.5 µg) and the viral NP gene (1.5 µg). Virus recovered from the cells was propagated in 10 day old embryonated chicken eggs and screened by western immuno-blot for expression of HA protein.

### Western Blot

Integral membrane proteins from infected cells were purified using a Mem-Per Kit (Thermo Scientific) as outlined by the protocol supplied by the manufacturer. Alternatively, cells were washed three times with ice cold PBS followed by gentle rocking in lysis buffer containing 50 mM Tris HCl, 150 mM NaCl, 0.25% deoxycholic acid, 1 mM EDTA, 1% NP40 and HALT protease inhibitor (Thermo Scientific). The lysate was processed by centrifugation at 14,000 x g and whole cell lysates were collected from the detergent soluble fraction. Sucrose purified virus, virus from allantoic fluid, or whole cell lysates were resolved in a 10% SDS acrylamide gel and transferred onto a nitrocellulose membrane. The membrane was incubated overnight at 4°C in 5% non-fat dry milk to block nonspecific proteins. GFP and β-actin were detected by incubation with goat anti-GFP (Rockland Immunochemical Inc. Gilbertsville, PA) followed by rabbit anti-goat IgG-HRP (Southern Biotech; Birmingham, AL) or mouse monoclonal antibody AC-40 (Sigma-Aldrich). HA immune-blot was detected by incubating the membrane with anti-sera from mice that survived three consecutive infections with live, PR/8 virus followed by incubation with anti-mouse Trublot (eBiosciences; San Diego, CA). Blots were developed using SuperSignal West Dura chemiluminesence substrate (Pierce; Rockford, IL). Western blots were imaged and pixel density was evaluated using FluorChem FC2 software (Alpha Inotech; San Leandro, CA).

### Flow Cytometry

CV-1 cells were cultured in DMEM in 100 units/ml penicillin and 100 µg/ml streptomycin, supplemented with 10% fetal calf serum. The cells were washed three times in PBS and PR/8, GP42-GFP or GP42-H1 were adsorbed onto the monolayer at MOI = 1 for 30 minutes at 37°C. The cells were cultured for 12 hours in virus growth medium containing DMEM supplemented with 7.5% bovine serum album and 2 µg/ml of TPCK-trypsin. Infected cells were washed three times in PBS and treated with 0.05% trypsin-EDTA. Cells were suspended in PBS containing 2% bovine serum albumin and Fc receptors were blocked by incubating with 2.4G2 antibodies (Becton Dickinson Biosciences; San Jose, CA). Cells were stained with mouse PR/8 antisera (1∶200 dilution) to detect surface expression of HA. Mouse antibodies were detected using phycoerythrin-conjugated goat anti-mouse IgG (Southern Biotech). Surface stained cells were fixed in 2% paraformaldehyde and data were acquired on an LSR II (Becton Dickinson) and analyzed using FlowJo software (Tree Star; Ashland, OR).

### Immunofluorescence Microscopy

CV-1 cells were cultured in DMEM containing 10% fetal calf serum on CC2 chamber slides (Thermo Scientific) overnight at 37°C in humidified atmosphere containing 5% CO_2_. The slides were washed in sterile PBS buffer and virus was adsorbed on the cells at a MOI = 10 for 1 hour. Unbound virus was removed by washing in PBS and cells were cultured an additional 20 hours in DMEM containing 7.5% bovine serum album and 2 µg/ml of TPCK-trypsin. Infected cells were washed in PBS and fixed in ice cold acetone for 10 min. Cells were blocked in 10% rabbit and goat serum for 30 min followed by incubation with mouse PR/8 anti-sera. Unbound antibodies were removed by washing three times in PBS containing 0.05% Tween20. The bound mouse antibodies were detected with goat anti-mouse IgG-TRITC (Southern Biotech; Birmingham, AL). Cells were washed and mounted with Prolong-Antifade containing DAPI (Invitrogen; Carlsbad, CA) and visualized using fluorescence microscopy. Images were captured using Spot Software (Diagnostic Instruments, Inc; Sterling Heights, MI).

### Enzyme-Linked ImmunoSorbent Assay (ELISA)

Sucrose purified PR/8 virus was suspended in PBS at 2 µg/ml and coated onto Immulon 4HBX plates (Thermo Fisher Scientific Inc) overnight at 4°C. Unbound PR/8 virus was removed by washing three times in PBS containing 0.05% Tween20 (PBS-T) and incubated with blocking buffer containing 2% BSA in PBS-T. Sera and secondary antibodies were diluted in blocking buffer and all incubations were for one hour at 37°C with three washes in PBS-T in between incubations. Influenza specific serum antibodies and standards were detected with anti-mouse IgG1-horseradish peroxidase (HRP), IgG2b-HRP (Southern Biotech; Birmingham, AL) or IgG2c-HRP (SeroTec; Raleigh, NC). 3,3′,5,5′ tetramethylbenzidine (TMB) substrate was added (Sigma Aldrich) and the reaction was stopped with an equal volume of 1 N HCl. Colorimetric substrate was detected by measuring the optical density at 450 nm. Antibody concentrations were determined by coating plates with goat anti-mouse IgG followed by incubation with mouse IgG1, IgG2b isotype controls (Sigma Aldrich) or IgG2c (Southern Biotechnology) isotype control followed by incubation with the corresponding HRP-conjugated secondary antibodies as described above. Serum antibody concentration is represented as relative units where 1 relative unit is defined as 1 ng/ml of the isotype control antibody.

### Hemagglutination Inhibition Assay

Hemagglutination inhibition (HAI) was performed as recommended by the WHO global influenza program and the Center for Disease Control [49]. In brief, sera were treated with receptor destroying enzyme (RDE, purchased from Denka Seiken; Tokyo, Japan) overnight at 37°C followed by RDE inactivation by incubation of the samples at 57°C for 30 minutes. Sera: RDE mixtures were serially diluted, incubated with four HA units of virus for one hour at room temperature. An equal volume of 0.5% washed chicken erythrocytes (Lampire Biological Laboratories; Pipersville, PA) was added and agglutination was observed after 30 min.

### Micro-neutralization

Micro-neutralization was performed as described [50] with some modifications. In brief, complement inactivated serum was serially diluted in DMEM growth media containing 100 u/ml penicillin, 100 µg/ml streptomycin, 2 mM L-glutamine, 25 mM HEPES buffer, 2 µg/ml TPCK-trypsin and 7.5% (w/v) bovine serum albumin and incubated with an equal volume containing 2×10^3^ TCID_50_ of PR/8 virus for one hour at 37°C in a 96-well tissue culture plate. 1.5×10^4^ Madin-Darby Canine Kidney (MDCK) cells (ATCC CCL-34) were added to the serum-virus mixture and incubated for 20 hours at 37°C in a humidified atmosphere containing 5% CO_2_. Cells were washed three times in PBS and fixed in 80% acetone. Endogenous peroxidases were quenched with 0.3% H_2_0_2_. All subsequent procedures are as described in the ELISA method. Viral replication was detected using anti-NP antibody (Abcam; Cambridge, MA) followed by detection with goat anti-rabbit HRP (Southern Biotechnology).

### Statistical Analysis

ELISA and HAI data were analyzed using GraphPad Prism software by performing a two-tailed unpaired t-test assuming unequal variance. Each data point represents samples collected from individual mice. Micro-neutralization was analyzed by performing non-linear regression analysis of a dose response curve. The Log of the 50% effective concentration (LogEC_50_) value was compared between two curves. Lung virus titers were analyzed by performing two-way ANOVA to compare multiple groups at various time-points. Bonferroni's post-test was used to generate P values for pairwise comparison. A P value less than 0.05 was considered statistically significant. Each experiment was repeated two additional times with similar results.
